# Identification of a hyperinflammatory sepsis phenotype using protein biomarker and clinical data in the ProCESS randomized trial

**DOI:** 10.1038/s41598-024-55667-5

**Published:** 2024-03-14

**Authors:** Kimberley M. DeMerle, Jason N. Kennedy, Chung-Chou H. Chang, Kevin Delucchi, David T. Huang, Max S. Kravitz, Nathan I. Shapiro, Donald M. Yealy, Derek C. Angus, Carolyn S. Calfee, Christopher W. Seymour

**Affiliations:** 1 Clinical Research, Investigation, and Systems Modeling of Acute Illness (CRISMA) Center, Pittsburgh, PA USA; 2 Division of Pulmonary, Allergy and Critical Care Medicine, University of Pittsburgh, Pittsburgh, PA USA; 3 Department of Critical Care Medicine, University of Pittsburgh, Pittsburgh, PA USA; 4 Department of Medicine, University of Pittsburgh, Pittsburgh, PA USA; 5 Department of Psychiatry, University of California San Francisco, San Francisco, CA USA; 6 Department of Emergency Medicine, University of Pittsburgh, Pittsburgh, PA USA; 7 Multidisciplinary Acute Care Research Organization (MACRO), Pittsburgh, PA USA; 8 Department of Emergency Medicine, Beth Israel Deaconess Medical Center, Boston, MA USA; 9 Center for Vascular Biology Research, Beth Israel Deaconess Medical Center, Boston, MA USA; 10 Division of Pulmonary and Critical Care Medicine, Department of Medicine and Anesthesia, University of California San Francisco, San Francisco, CA USA; 11 Present Address: Division of Pulmonary, Allergy and Critical Care Medicine, University of Pittsburgh, 3459 Fifth Avenue, NW628, Pittsburgh, PA 15213 USA

**Keywords:** Sepsis, Phenotypes, Biomarkers, Prognostic markers, Machine learning

## Abstract

Sepsis is a heterogeneous syndrome and phenotypes have been proposed using clinical data. Less is known about the contribution of protein biomarkers to clinical sepsis phenotypes and their importance for treatment effects in randomized trials of resuscitation. The objective is to use both clinical and biomarker data in the Protocol-Based Care for Early Septic Shock (ProCESS) randomized trial to determine sepsis phenotypes and to test for heterogeneity of treatment effect by phenotype comparing usual care to protocolized early, goal-directed therapy(EGDT). In this secondary analysis of a subset of patients with biomarker sampling in the ProCESS trial (n = 543), we identified sepsis phenotypes prior to randomization using latent class analysis of 20 clinical and biomarker variables. Logistic regression was used to test for interaction between phenotype and treatment arm for 60-day inpatient mortality. Among 543 patients with severe sepsis or septic shock in the ProCESS trial, a 2-class model best fit the data (p = 0.01). Phenotype 1 (n = 66, 12%) had increased IL-6, ICAM, and total bilirubin and decreased platelets compared to phenotype 2 (n = 477, 88%, p < 0.01 for all). Phenotype 1 had greater 60-day inpatient mortality compared to Phenotype 2 (41% vs 16%; p < 0.01). Treatment with EGDT was associated with worse 60-day inpatient mortality compared to usual care (58% vs. 23%) in Phenotype 1 only (p-value for interaction = 0.05). The 60-day inpatient mortality was similar comparing EGDT to usual care in Phenotype 2 (16% vs. 17%). We identified 2 sepsis phenotypes using latent class analysis of clinical and protein biomarker data at randomization in the ProCESS trial. Phenotype 1 had increased inflammation, organ dysfunction and worse clinical outcomes compared to phenotype 2. Response to EGDT versus usual care differed by phenotype.

## Introduction

Sepsis is common and deadly, accounting for up to one sixth of hospital admissions^1–3^ and more than 19 million cases annually worldwide^1,2^. Despite advances in the understanding of the biology and immune response in sepsis, significant controversy remains regarding the best approach to sepsis treatment. Most trials over the past decade investigating new sepsis treatments have not found a mortality benefit to specific early and aggressive resuscitation approaches, including the Protocolized Care for Early Septic Shock (ProCESS) trial^4^. Heterogeneity in host response, pathogen, and organ dysfunction in sepsis may explain the results of recent resuscitation trials. Patients that share clinical or biologic characteristics, termed phenotypes, may respond differently to treatment. Trials that report average treatment effects ignore this heterogeneity, even when explored in traditional subgroup analyses of single risk factors.

The Sepsis ENdotyping in Emergency CAre (SENECA) study found 4 sepsis phenotypes using routinely available clinical data at presentation to the emergency department (ED)^5^. Phenotypes were distinct from traditional subgroups by illness severity and differed in severity, laboratory abnormalities, organ dysfunction patterns, and short- and long-term outcomes. However, potentially important protein biomarkers were not included in the derivation of the phenotypes. Such biomarkers, including inflammatory cytokines, and markers of endothelial dysfunction or abnormal coagulation, contribute to phenotypes in acute respiratory distress syndrome, pancreatitis, and other acute conditions, yet have an unknown role in sepsis classification^6–8^.

We sought to determine sepsis phenotypes using clinical and biomarker data with unsupervised machine learning in the ProCESS trial, correlate these phenotypes with clinical outcomes, and assess for differential treatments effects by biomarker-based phenotypes. Protein biomarkers used in this analysis, that were not used in the derivation of the SENECA phenotypes, include interleukin-6 (IL-6), Plasminogen Activator Inhibitor-1 (PAI-1), and Intercellular Adhesion Molecule (ICAM). These biomarkers were used to create a phenotyping approach that is different from the SENECA phenotypes.

## Methods

This project involved 3 steps. First, we used latent class analysis on protein biomarkers combined with clinical data to inform phenotypes in the ProCESS trial. Second, we explored the correlation of the sepsis phenotypes with a variety of sepsis biomarker variables that reflect changes in inflammation, coagulation, and endothelial function. We examined phenotype association with clinical outcomes, including admission to intensive care, vasopressor use and mechanical ventilation use, any-cause 60-day inpatient mortality and any-cause 365-day mortality. Third, we tested for differential treatment effects by phenotype by using regression models to evaluate for statistical interactions between study arm (early, goal directed therapy (EGDT) vs. usual care) and sepsis phenotype.

### Data

Clinical and biomarker data was obtained from the ProCESS trial^4^. ProCESS enrolled 1341 patients with septic shock who were randomized 1:1:1 to protocol-based EGDT (N = 439), protocol-based standard care (N = 446) or usual care (N = 456) at 31 centers from 2008 to 2013. The primary outcome, 60-day inpatient mortality, is the same as the primary outcome of the original ProCESS trial. 60-day inpatient mortality captures inpatient mortality from any-cause at 60 days. We restricted our analysis to the subset of patients with data available for the biomarkers IL-6, PAI-1, and ICAM. Biomarker acquisition was predetermined on a subset of patient within financial limitations. The excluded cohort of patients was similar to the primary cohort (Table S1). Protocolized standard care was excluded from treatment effect models to be consistent with both the SENECA analysis of the ProCESS trial and the Protocolized Resuscitation in Sepsis Meta-Analysis (PRISM) meta-analysis, a harmonized dataset of patient- level data from 3 large randomized controlled trials in EGDT that investigated the treatment effect of protocolized EGDT versus usual care^9^.

### Clinical and biomarkers for latent class analysis

We selected 20 clinical and biomarker variables as candidates for phenotyping based upon prior models in the SENECA study and those that contributed to identification of a hyperinflammatory phenotype in acute respiratory distress syndrome (ARDS)^5,7^. Variables included age, vital signs (heart rate, respiratory rate, systolic blood pressure, temperature, body mass index (BMI)), markers of organ dysfunction or inflammation (creatinine, total bilirubin, platelet count, white blood cell count, urine output, glucose, albumin), organ support prior to randomization (mechanical ventilation, vasopressor use). Additional laboratory values included were serum sodium and hematocrit. Baseline, pre-randomization values for IL-6, PAI-1, and ICAM were included, while we did not include others such as von Willebrand factor, Surfactant Protein D and soluble tumor necrosis factor-1 due to high missingness (> 75%) (Table S2). Multiple measurements prior to randomization occurred in approximately 10% of the variables. When multiple measurements occurred, the nearest value prior to randomization was used for analysis. Full information maximum likelihood was used for missing data; no multiple imputation was used for missing data as the latent class procedure is robust to missingness^10^_._

### Correlation with clinical outcomes and differential treatment effects

To understand the correlation between phenotypes and biomarkers of host response, we studied biomarkers measured at baseline but not included in latent class as they may be hypothesis generating and reflect the underlying biology of the phenotypes. These biomarkers included angiopoietin-2 (Ang-2), prothrombin, E-selectin, tumor necrosis factor (TNF), interleukin-10 (IL-10), C-reactive protein, and D-dimer. While the latter six were previously studied in the SENECA analysis, Ang-2 was unique to this analysis^5^. The primary clinical outcome measured in all 543 patients was 60-day inpatient mortality. Other clinical outcomes were hospital length of stay, intensive care use (ICU) and length of stay, and intravenous fluid volume (post-randomization). The test for heterogeneity of treatment effect by phenotype was restricted to between patients who received protocolized EGDT (N = 185) and usual care (N = 179).

### Statistical analysis

To derive the sepsis phenotypes, we assessed candidate variable missingness, distributions, and correlation. We excluded variables with a high degree of missingness, standardized the variables and used log transformation of non-normally distributed variables. For variables that were either below or above the limit of detection, we replaced with value with a value that was 0.5 and 2 times the lower and upper limit of detection, respectively. We assessed for outliers clinically and removed if appropriate, which occurred in less than 0.3% of variables. We evaluated correlation in order to inform sensitivity analysis of highly correlated variables (rho > 0.5). We used latent class analysis to derive the phenotypes on all 543 patients. Latent class analysis is a well-validated statistical model-based technique that identifies latent, or unobserved, classes within a population agnostic to outcome or treatment variables^11^. To determine the optimal number of phenotypes (k), we evaluated the Bayesian Information Criterion (BIC, preferable if lower), entropy (preferable if near 1.0), Vuong-Lo-Mendell-Rubin (VLMR) p-value, and class size (number of patients per phenotype). After assigning each patient a phenotype based on the greatest posterior probability of membership, we investigated distributions of probabilities for assigned and unassigned phenotypes. We used logistic regression to test heterogeneity of treatment effect by phenotype, considering a significant test of interaction for p < 0.05. Data was presented as mean (SD) or median [IQR], as appropriate.

Data analysis used Stata 15.1 (StataCorp, College Station, Texas) and Mplus 8.2 (Muthen and Muthen).

### Ethics approval and consent to participate

The randomized controlled trial, A Randomized Trial of Protocol-Based Care for Early Septic Shock (ProCESS) (NCT00510835) was approved by the University of Pittsburgh Institutional Review Board and overseen by the Data Safety and Monitoring Board, and all methods were performed in accordance with their relevant guidelines and regulations. All study participants or their legal representatives provided written informed consent.

## Results

Among 543 eligible patients, most were male (59.5%) with more than 2 co-morbidities (mean Charlson score 2.7 (SD 2.7)) (Table 1). The mean APACHE III score was 62 (SD 23) and the median lactate was 2.5 mmol/L (IQR 1.4–4.3 mmol/L). Nearly 90% of patients were admitted to the ICU with a mean ICU length of stay of 5 days (SD 5 days). One in five patients required vasopressors or mechanical ventilation (pre-randomization).

**Table 1:** Baseline characteristics by phenotype in the ProCESS randomized trial (N = 543).

Variable	Overall (n = 543)	Phenotype 1 (n = 66, 12%)	Phenotype 2 (n = 477, 88%)
Demographics			
Age, years, mean (SD)	60 (16)	61 (13)	60 (16)
Gender, no. (%)			
Male	323 (59.5%)	47 (71.2%)	276 (57.9%)
Female	220 (40.5%)	19 (28.8%)	201 (42.1%)
Race, no. (%)			
White	380 (70.0%)	44 (66.7%)	336 (70.4%)
Black	121 (22.3%)	15 (22.7%)	106 (22.2%)
Other	42 (7.7%)	7 (10.6%)	35 (7.3%)
Charlson comorbidity Index, mean (SD)	2.7 (2.7)	3.2 (2.7)	2.7 (2.7)
Glasgow Coma Score, mean (SD)	13.6 (3.0)	13.7 (2.9)	13.6 (3.0)
Apache III score, mean (SD)	61.7 (23.0)	69.6 (22.2)	60.6 (22.9)
Variables in phenotype model			
Albumin, g/dL, mean (SD)	3.1 (0.8)	2.7 (0.8)	3.1 (0.8)
BMI, kg/m^2^, mean (SD)	28.2 (7.8)	27.8 (7.3)	28.2 (7.9)
Serum creatinine, mg/dL, mean (SD)	2.3 (2.0)	2.1 (1.4)	2.3 (2.0)
Glucose, mg/dL, mean (SD)	163 (119)	111 (54)	170 (124)
Heart rate, beats/min, mean (SD)	113 (24)	116 (23)	112 (24)
Hematocrit, %, mean (SD)	35 (7)	31 (7)	36 (7)
Platelet count, in thousands, mean (SD)	229 (138)	73 (55)	250 (133)
Respiratory rate, resps/min, mean (SD)	23 (7)	22 (7)	23 (7)
Systolic blood pressure, mmHg, mean (SD)	100 (28)	102 (30)	100 (28)
Sodium, mEq/L, mean (SD)	136 (7)	135 (7)	136 (6)
Temperature, °C, mean (SD)	37.4 (1.6)	37.2 (1.7)	37.4 (1.6)
Total bilirubin, mg/dL, mean (SD)	1.4 (1.9)	4.8 (3.5)	1.1 (1.1)
WBC count, in thousands, mean (SD)	15 (9)	5 (6)	17 (9)
Vasopressors, no. (%)	104 (19%)	15 (23%)	89 (19%)
Mechanical ventilation, no. (%)	87 (16%)	12 (18%)	75 (16%)
Urine output, mL/h, median [IQR]	11 [0–69]	8 [0–50]	12 [0–70]
ICAM, ng/mL, median [IQR]	526 [332–851]	1320 [607–2425]	466 [310–683]
IL-6, pg/mL, median [IQR]	344 [87–3003]	4841 [419–57,464]	281 [77–1774]
PAI-1, ng/mL, median [IQR]	15 [8–27]	14 [8–37]	15 [8–27]

*SD* standard deviation, *BMI* body mass index, *IQR* interquartile range, *ICAM* intracellular adhesion molecule, *IL-6* interleukin-6, *PAI-1* plasminogen activator inhibitor-1.

### Derivation of clinical and biomarker phenotypes

Latent class analysis suggested that a 2-class model provided a significant improvement in model fit as compared to one class model (VMLR p = 0.01, Table S3) There was no evidence that adding additional classes improved model fit (Table S3, Fig. S1). In the final model, phenotype 1 had 66 patients (12%) and phenotype 2 had 477 patients (88%). The posterior probability of phenotype membership assigned phenotypes was high (phenotype1 = 0.90, SD 0.15; phenotype 2 = 0.98, SD 0.06) (Fig. S2). In sensitivity analyses where the highly correlated variables albumin, heart rate, urine output were removed, model fit was largely similar (Table S4).

### Clinical characteristics, biomarkers, and outcomes by phenotypes

**Figure 1:**
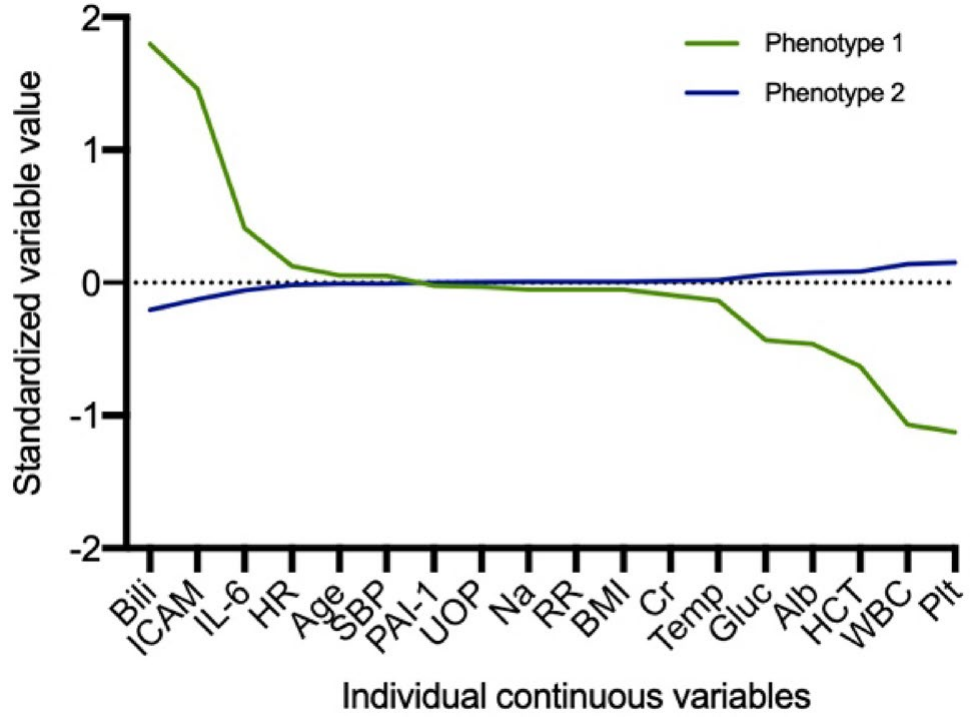
Phenotype variables ranked by the difference in mean standardized value. Mean standardized difference of continuous variables comparing Phenotype 1 (green) and Phenotype 2 (blue). The variables are ranked on the x-axis by degree of separation from Phenotype 1 versus 2 with maximum positive degree of separation on the right to maximum negative degree of separation on the left. *Bili* bilirubin, *ICAM* intercellular adhesion molecule, *IL-6* interlukin-6, *HR* heart rate, *SBP* systolic blood pressure, *PAI-1* plasminogen activator inhibitor-1, *RR* respiratory rate, *BMI* body mass index, *Temp* temperature, *HCT* hematocrit, *WBC* white blood cell.

**Figure 2:**
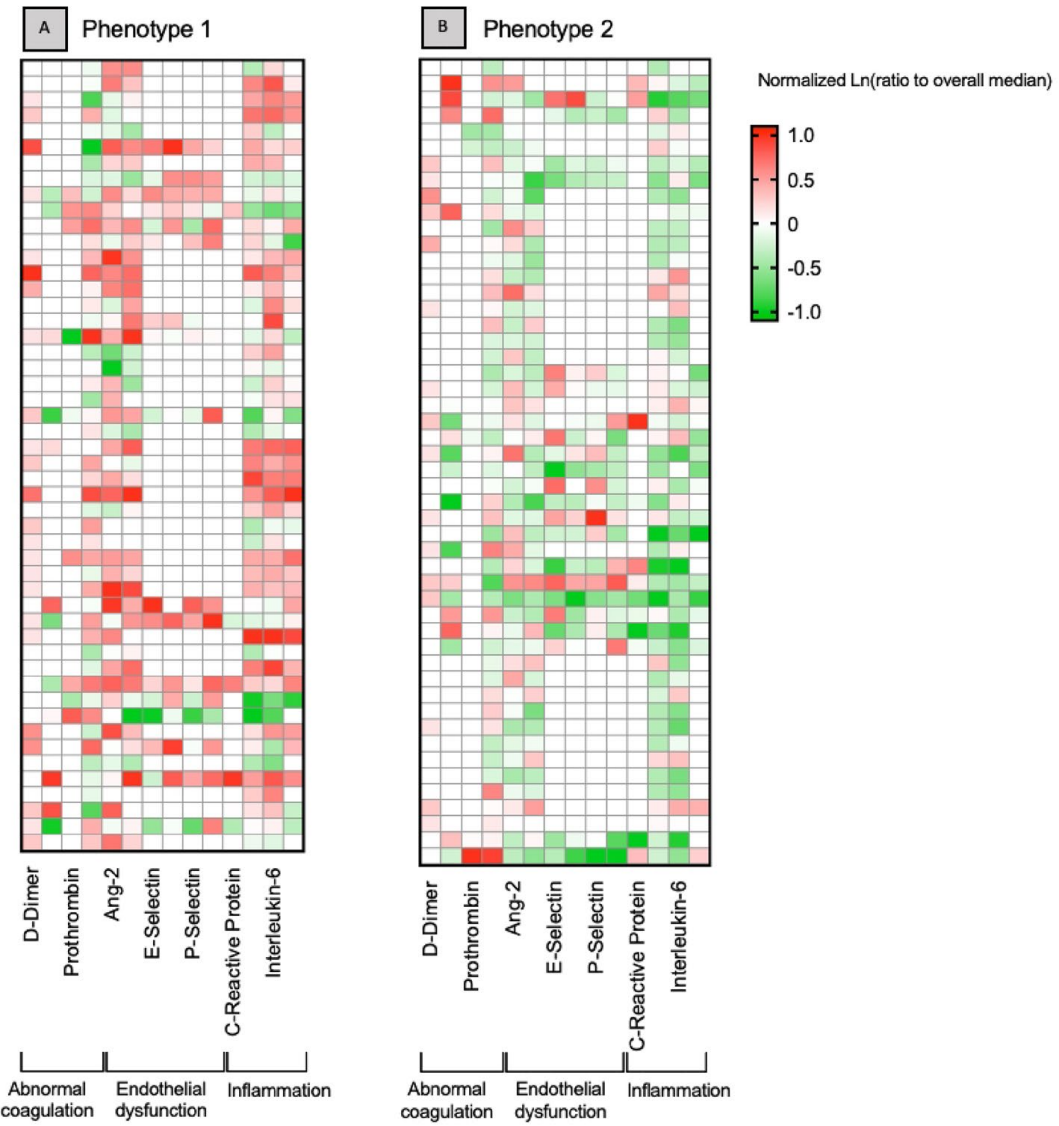
Heatmap of biomarkers by phenotype (N = 100). Heatmap showing the log of the fold change of the median biomarker value (column) per patient (row) for various markers of the septic host response grouped by those reflecting coagulation, endothelium and inflammation in a random selection of 100 patients from (**A**) phenotype 1 and (**B**) phenotype 2. Red represents greater median biomarker value for that phenotype compared to the median of the entire study, while green represents lower values of the biomarker compared to the median of the entire study. White cells are those in which the biomarker was not measured.

The 2 phenotypes had distinct clinical characteristics. For example, compared to phenotype 2, phenotype 1 had increased bilirubin and decreased white blood cell count and platelets (Fig. 1, Table 1). The 2 phenotypes also had distinct biomarker profiles, amongst the biomarkers not included in the latent class model. Phenotype 1 had increased levels of biomarkers reflecting inflammation, endothelial dysfunction and abnormal coagulation. For example, TNF (124 [349–287] vs. 20 [13–69]) and IL-10 (101 [22–966] vs. 35 [17–91]) were greater in phenotype 1 versus phenotype 2, respectively (p < 0.01 for both) (Fig. 2, Table S5).

**Figure 3:**
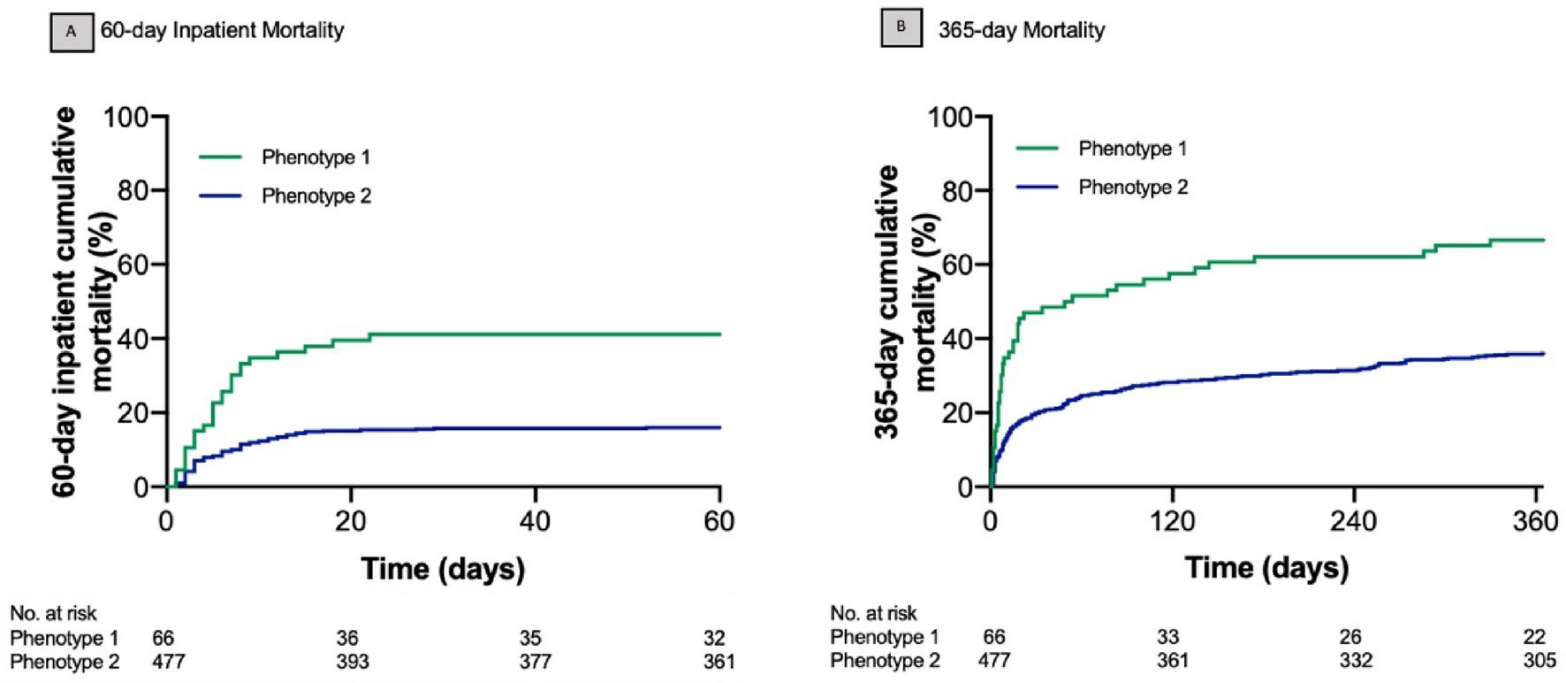
Short- and long-term mortality by phenotype (N = 543). (**A**) 60-day inpatient mortality probability. (**B**) 365-day mortality probability, by phenotype, where phenotype 1 is green and phenotype 2 is blue. Both panels show significant differences in mortality probability by phenotype (log rank P < 0.01). Panel (**A**) captures inpatient mortality from any-cause at 60 days whereas Panel (**B**) captures overall mortality from any cause up to 365 days.

The phenotypes were prognostic of clinical outcomes. More patients were admitted to the intensive care unit in phenotype 1 compared to phenotype 2 (97% versus 88%, p = 0.03, Table 2), and 60-day inpatient mortality was greater in phenotype 1 (41% vs. 16%, p < 0.01, Fig. 3).

**Table 2:** Clinical outcomes by phenotype (N = 543).

Outcomes	Overall (n = 543)	Phenotype 1 (n = 66, 12%)	Phenotype 2 (n = 477, 88%)	p-value
Admission to intensive care unit, no. (%)	485 (89%)	64 (97%)	421 (88%)	0.03
Intensive care length of stay, days, mean (SD)	5 (5)	6 (6)	4 (4)	0.06
Intravenous fluid (post random.), mL, mean (SD)	2816 (2099)	3207 (1964)	2763 (2114)	0.03
60-day inpatient mortality, no. (%)	103 (19%)	27 (41%)	76 (16%)	< 0.01
90-day mortality, no. (%)	163 (30%)	36 (55%)	127 (27%)	< 0.01
365-day mortality, no. (%)	216 (40%)	44 (67%)	172 (36%)	< 0.01

### Differential treatment effects by phenotype

**Figure 4:**
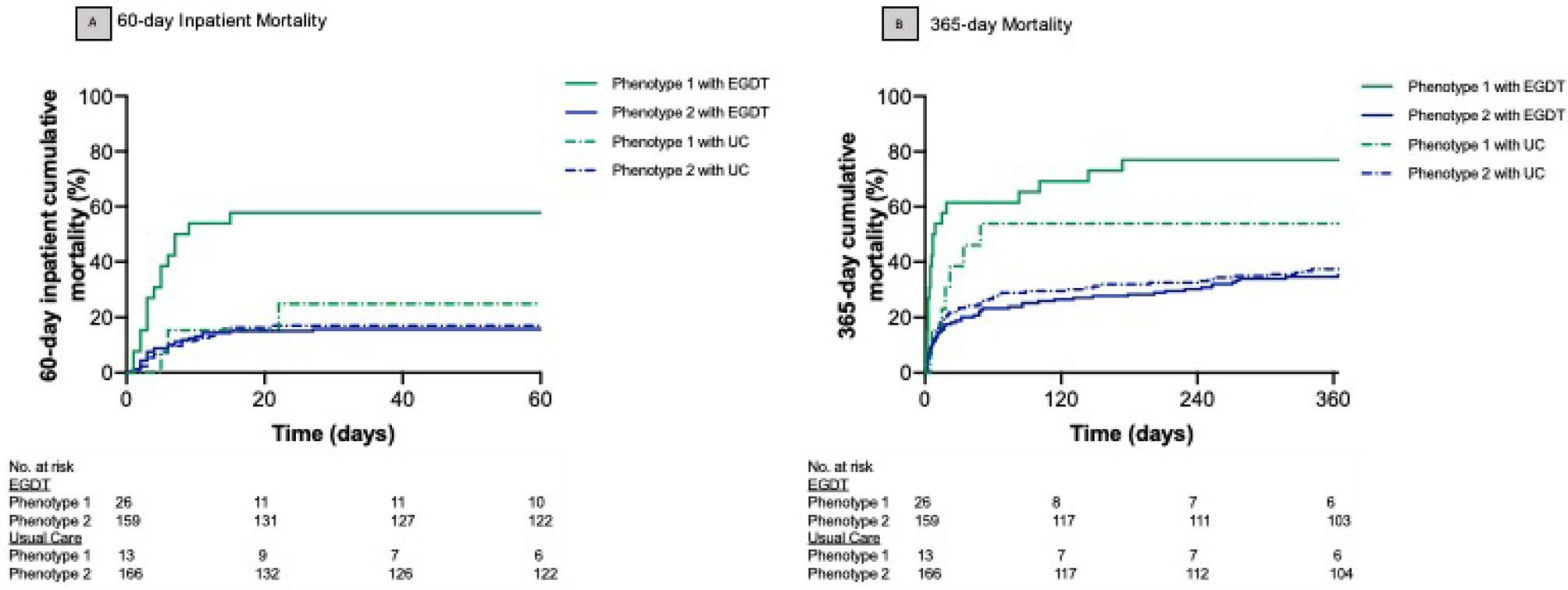
Short- and long-term mortality stratified by phenotype and treatment arm (N = 364). (**A**) 60-day inpatient mortality probability. (**B**) 365-day mortality probability, by phenotype and treatment arm, where phenotype 1 is green, phenotype 2 is blue, EGDT is a solid line, Usual Care (UC) is a dashed line. Panel (**A**) captures inpatient mortality from any cause at 60 days whereas Panel (**B**) captures overall mortality from any cause up to 365 days.

The treatment effect analysis was completed on 364 patients (n = 185 with EGDT and N = 179 with usual care). Balance of baseline covariates was preserved comparing treatment arms within phenotype (Table S6). In phenotype 1, treatment with protocolized EGDT was associated with worse 60-day inpatient mortality compared to usual care (58% vs. 23%), and not associated with outcome in phenotype 2 (16 vs. 17%, p-value for interaction = 0.05). There was no differential treatment effect with protocolized EGDT versus usual care by phenotype at 365 days (p-value for interaction = 0.13, Fig. 4, Table S7). In a sensitivity analysis, we tested for evidence of differential treatment effects by severity of illness. The mean APACHE III score was greater in phenotype 1 compared to phenotype 2 (70 (SD 22) versus 61 (SD 23), p = 0.003 (Table 1). There was no interaction between continuous APACHE III score and treatment with EGDT vs. usual care for 60-day inpatient mortality or 365-day mortality (p-values for interaction = 0.42 and p = 0.48, respectively, Fig. S3, Table S8).

### Comparison to SENECAsepsis phenotypes

The hyperinflammatory phenotype 1 (N = 88, 12%) was similar to ProCESS patients with the delta clinical phenotype identified in the recent SENECA study (N = 89, 16%, Table S9). Phenotype 1 and the SENECA delta-type patients both had elevated serum lactate, total bilirubin, reduced platelets, and poor clinical outcomes (Table S9). We observed that biomarkers ICAM and IL-6 were higher in phenotype 1 and the delta SENECA phenotype, compared to phenotype 2 and non-delta SENECA phenotypes (p < 0.01 for both, Table S10).

## Discussion

In the ProCESS randomized trial, 2 sepsis phenotypes were identified using clinical and biomarker variables before randomization. The phenotypes had distinct clinical and biomarker profiles and were prognostic of clinical outcomes. Phenotype 1 was characterized by increased inflammation and organ dysfunction and had worse clinical outcomes. Response to EGDT versus usual care differed by phenotype.

Our work extends the observations of the recent SENECA study that proposed clinical sepsis phenotypes (α, ß, y, and ∂) using routinely available data at presentation in the electronic health record, and validated these phenotypes in three clinical trials including ProCESS. These phenotypes were distinctfrom traditional subgroups by illness severity and organ failure burden, differed in laboratory abnormalities, were prognostic of clinical outcomes, and, in computer simulation, had differential treatment effects by phenotype^5^. We extend these results, by demonstrating that biomarkers are important in phenotype derivation. This finding is similar to how biomarker data is used in ARDS, where protein biomarkers are key to a hyperinflammatory phenotype, with elevated IL-6 and ICAM-1^7^. By including biomarkers in sepsis derivation models, these new findings refine previously published clinical phenotypes and found new heterogeneity of treatment effect for EGDT. This analysis is novel in its use of both protein biomarkers and clinical data in the derivation of sepsis phenotypes.

The addition of protein biomarkers to clinical data confirmed a sepsis phenotype at the highest risk for poor outcomes and greater inflammatory biomarkers. Termed Phenotype 1, these patients resemble those found in the ∂ SENECA group. Both Phenotype 1 and the ∂ phenotype were the least frequent and the most deadly, exhibiting similar patterns of inflammation, abnormal coagulation, and endothelial dysfunction. Future work that extends the integration of phenotypes beyond clinical and protein data to molecular markers from the transcriptome could further refine this hyperinflammatory phenotype^12,13^. Current clinical, biomarker, and transcriptomic phenotyping strategies are not correlated, highlighting the need for future complementary approaches in precision medicine for sepsis^14^. Such work will require a balance between mechanistic discovery and practical classification at the bedside.

This study confirmed that response to sepsis resuscitation approaches differs by phenotype. The data extends previous in silico models in SENECA, which suggested the ProCESS trial would more often conclude for harm if the proportion of delta patients enrolled was increased^5^. Although the mechanism is unclear, further study of the biologic mediators in treatment-related harm in specific patients is warranted. Future trial designs could consider the identification of sepsis phenotypes at enrollment, potentially enriching for specific phenotypes and treatment combinations.

This study has several limitations. First, this was a secondary analysis of a randomized controlled trial, which limits treatment conclusions until confirmed prospectively; however, these results can be used to inform inclusion criteria in future trials on a more personalized approach to sepsis resuscitation. Second, the decision to exclude the protocolized standard care arm from the treatment interaction model was post-hoc; however, this is consistent with the SENECA analysis of the ProCESS trial and the PRISM meta-analysis. Third, there are many protein biomarkers to consider for sepsis phenotyping. We chose those markers proposed in prior work and available in the ProCESS trial^7^. Fourth, missing data was present in the trial dataset. However, this dataset has less missingness than other electronic health record analyses, where variable missingness can approach 90% HER analysis other studies^5^. Furthermore, we used latent class analysis for clustering, a method robust to missing data^15,16^. Fifth, patients enrolled in this subset of the ProCESS randomized trial may not be generalizable to other sepsis cohorts, and continued assessment of reproducibility is warranted. Sixth, we acknowledge that phenotype 1 was a small proportion of the trial dataset (12%). However, phenotype size is known to be variable and phenotype 1 size is similar to the frequency of sepsis subclasses derived in the Molecular Diagnosis and Risk Stratification of Sepsis (MARS) cohort (34–41%), SENECA study (13–33%), and Recombinant Human Activated Protein C Worldwide Evaluation in Severe Sepsis (PROWESS) trial (4–22%)^5,12,17^.

## Conclusions

We used latent class analysis to identify two severe sepsis phenotypes with distinct clinical and biomarker profiles in the ProCESS trial. Phenotype 1 has increased inflammation, organ dysfunction and worse clinical outcomes. Response to EGDT versus usual care differed by phenotype. Treatment with protocolized EGDT was associated with worse 60-day inpatient mortality in phenotype 1 compared to usual care, and not associated with change in outcomes in phenotype 2.

### Supplementary Information

Supplementary Information.

## Data Availability

The datasets used and/or analyzed during the current study are available from the corresponding author upon reasonable request.
