# Supplementary material for: Identification of a hyperinflammatory sepsis phenotype using protein biomarker and clinical data in the ProCESS randomized trial
Source: Sci Rep. 2024 Mar 14;14:6234. doi: 10.1038/s41598-024-55667-5 (PMC10940677; doi:10.1038/s41598-024-55667-5)
Supplement: Supplementary file 1 — Supplementary Information. [file 41598_2024_55667_MOESM1_ESM.docx]

**Sepsis phenotyping using clinical and biomarker data in the ProCESS randomized trial**

**Figure S1**. Bayesian information criterion and entropy statistics from latent class analysis

**Figure S2.** Probabilities of phenotype assignment for phenotype membership

**Figure S3**. Short- and long-term mortality stratified by Apache III score and treatment arm (EGDT vs. usual care, N=543)

**Table S1.** Comparison of primary cohort (n=543) versus cohort excluded for missing IL-6 (n=798)

**Table S2**. Assessment of missing data for variables used in phenotype model

**Table S3.** Results from latent class analysis for clinical and biomarker data

**Table S4.** Results from latent class analysis sensitivity analysis removing the highly correlated variables albumin, heart rate and urine output

**Table S5.** Biomarkers variables not included in latent class analysis (N=543)

**Table S6.** Post-randomization processes of care by phenotype and treatment arm (N=364)

**Table S7.** Patient characteristics by treatment arm and phenotype (N=364)

**Table S8.** Treatment effect on clinical outcomes by phenotype (N=364)

**Table S9.** Short- and long-term outcomes for treatment by APACHE III score (N=364)

**Table S10.** Clinical characteristics and outcomes of phenotype 1 and SENECA delta phenotype

**Table S11.** Biomarker comparison in clinical + biomarker phenotypes to clinical only phenotypes

**Figure S1**. Bayesian information criterion and entropy statistics from latent class analysis


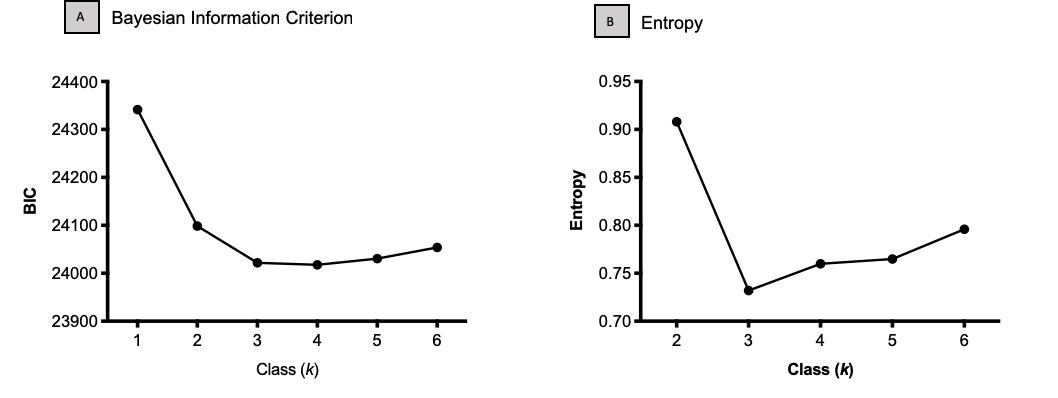


(A) Bayesian Information Criterion (BIC) across class k=1 to k=6, where lower BIC suggests more optimal fit. (B) Entropy across class k=1 to k=6, where higher entropy suggests higher degree of class separation

**Figure S2.** Probabilities of phenotype assignment for phenotype membership


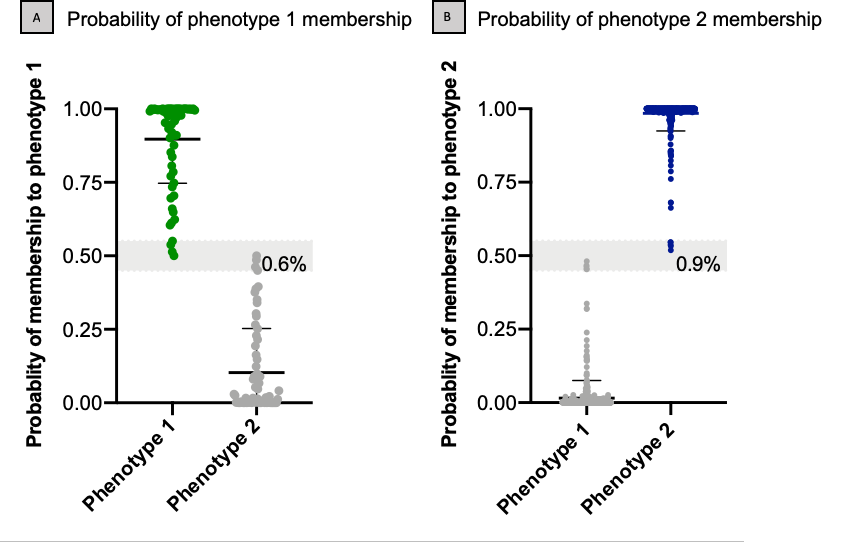


(A) Probabilities of assignment to phenotype 1, and green for those actually assigned to phenotype 1, (B) Probabilities for patients assigned to phenotype 2, and blue for those actually assigned to beta. Black lines correspond to median [IQR] of probability. Gray shading corresponds to region with a 45-55% (low or marginal) probability of assignment. Inset proportion is the percentage of 543 patients in the marginal region.

**Figure S3**. Short- and long-term mortality stratified by Apache III score and treatment arm (EGDT vs. usual care, N=543)


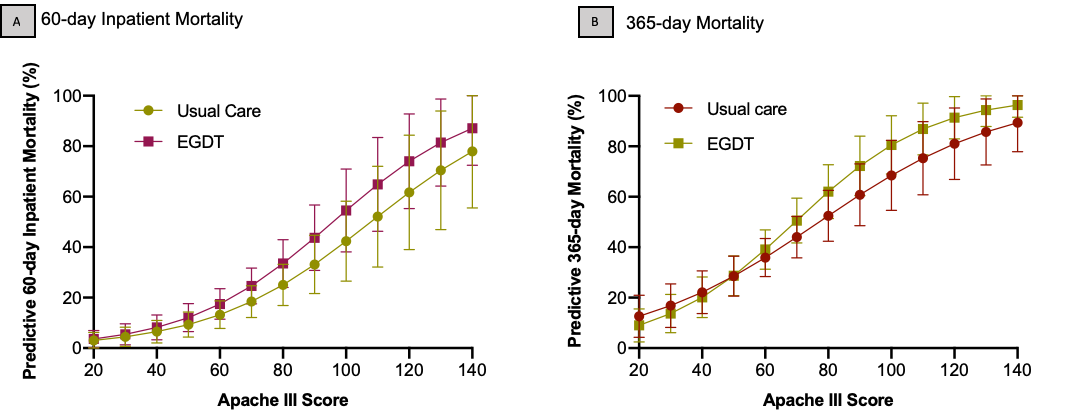


(A) Predicted 60-day inpatient mortality and (B) predicted 60-day inpatient mortality by Apache III score and treatment group, where Usual care is yellow and EGDT is red. The bars represent the bars 95% confidence interval.

**Table S1.** Comparison of primary cohort (n=543) versus cohort excluded for missing IL-6 (n=798)

| **Variable** | | **Overall (n=543)** | **Excluded cohort (n=798)** |
| --- | --- | --- | --- |
| **Demographics** | |  |  |
|  | Age, years, mean (SD) | 60 (16) | 61 (16) |
|  | Gender, no. (%) |  |  |
|  | Male | 323 (59.5%) | 425 (53.3%) |
|  | Female | 220 (40.5%) | 373 (46.7%) |
|  | Race, no. (%) |  |  |
|  | White | 380 (70.0%) | 532 (66.7%) |
|  | Black | 121 (22.3%) | 211 (26.4%) |
|  | Other | 42 (7.7%) | 55 (6.9%) |
|  | Charlson Comorbidity Index, mean (SD) | 2.7 (2.7) | 2.6 (2.6) |
|  | Apache III score, mean (SD) | 61.7 (23.0) | 60.7 (22.3) |
| **Treatment Arm** | |  |  |
|  | Early, goal-directed therapy | 185 (34%) | 254 (32%) |
|  | Protocolized standard care | 179 (33%) | 267 (33%) |
|  | Usual care | 179 (33%) | 277 (35%) |
| **Variables in phenotype model** | |  |  |
|  | Albumin, g/dL, mean (SD) | 3.1 (0.8) | 3.2 (0.9) |
|  | BMI, kg/m^2^, mean (SD) | 28.2 (7.8) | 28.4 (7.7) |
|  | Serum Creatinine, mg/dL, mean (SD) | 2.3 (2.0) | 2.3 (1.9) |
|  | Glucose, mg/dL, mean (SD) | 163 (119) | 160 (110) |
|  | Heart Rate, beats/min, mean (SD) | 113 (24) | 110 (23) |
|  | Hematocrit, %, mean (SD) | 35 (7) | 36 (7) |
|  | Platelet Count, in thousands, mean (SD) | 229 (138) | 233 (135) |
|  | Respiratory Rate, resps/min, mean (SD) | 23 (7) | 23 (7) |
|  | Systolic Blood Pressure, mmHg, mean (SD) | 100 (28) | 101 (29) |
|  | Sodium, mEq/L, mean (SD) | 136 (7) | 136 (6) |
|  | Temperature, °C, mean (SD) | 37.4 (1.6) | 37.4 (1.4) |
|  | Total Bilirubin, mg/dL, mean (SD) | 1.4 (1.9) | 1.5 (2.2) |
|  | WBC Count, in thousands, mean (SD) | 15 (9) | 15 (9) |
|  | Vasopressors, no. (%) | 104 (19%) | 124 (16%) |
|  | Mechanical Ventilation, no. (%) | 87 (16%) | 116 (15%) |
|  | Urine Output, mL/hr, Per Hour, no. (%) | 11 [0 - 69] | 5 [0 - 60] |
|  | ICAM, ng/mL, median [IQR] | 526 [332 - 851] | 523 [366 - 705] |
|  | IL-6, pg/mL, median [IQR] | 344 [87 - 3003] | - |
|  | PAI-1, ng/mL, median [IQR] | 15 [8 - 27] | 13 [7 - 35] |

*Abbreviations:* SD: standard deviation; BMI: body mass index; IQR: interquartile range; ICAM: intracellular adhesion molecule; IL-6: interleukin-6; PAI-1: plasminogen activator inhibitor-1

**Table S2**. Assessment of missing data for variables used in phenotype model

| **Variable** | **Overall Missingness** | **Phenotype 1 (n=66)** | **Phenotype 2 (n=477)** | **Range** |
| --- | --- | --- | --- | --- |
| Age, years | 11 (2%) | 0 (0%) | 11 (2%) | 19 - 89 |
| Albumin, g/dL | 174 (32%) | 15 (23%) | 159 (33%) | 0.4 - 6.1 |
| BMI, kg/m^2^ | 18 (3%) | 1 (2%) | 19 (4%) | 12.68 - 79.72 |
| Serum Creatinine, mg/dL | 29 (5%) | 2 (3%) | 29 (6%) | 0.4 - 17.3 |
| Glucose, mg/dL | 26 (5%) | 2 (3%) | 26 (5%) | 2 - 1342 |
| Heart Rate, beats/min | 0 (0%) | 0 (0%) | 2 (0.4%) | 32 - 226 |
| Hematocrit, % | 21 (4%) | 2 (6%) | 17 (4%) | 15 - 63 |
| Platelet Count, in thousands | 24 (4%) | 4 (6%) | 21 (4%) | 4 - 1102 |
| Respiratory Rate, respirations/minute | 1 (0.2%) | 0 (0%) | 1 (0.2% | 5 - 60 |
| Systolic Blood Pressure, mmHg | 0 (0%) | 0 (0%) | 0 (0%) | 40 - 234 |
| Sodium, mEq/L | 24 (4%) | 1 (2%) | 23 (5%) | 101 - 161 |
| Temperature, °C | 5 (0.9%) | 0 (0%) | 6 (1%) | 28.8 - 41.4 |
| Total Bilirubin, mg/dL | 289 (53%) | 40 (61%) | 249 (52%) | 0.1 - 12.7 |
| WBC Count, in thousands | 23 (4%) | 6 (9%) | 22 (5%) | <1 - 99 |
| Vasopressors, (%) | 0 (0%) | 0 (0%) | 0 (0%) | Yes - No |
| Mechanical Ventilation (%) | 0 (0%) | 0 (0%) | 0 (0%) | Yes - No |
| Urine Output, mL/hr | 6 (1%) | 2 (3%) | 4 (0.8%) | 0 - 1,117 |
| PAI-1, ng/mL | 392 (72%) | 54 (81%) | 338 (71%) | 1.5 - 153.5 |
| ICAM, ng/mL | 353 (65%) | 51 (77%) | 302 (63%) | 28.61 - 4053.09 |
| IL-6, pg/mL | 0 (0%) | 0 (0%) | 0 (0%) | 6 - 2,935,380 |

*Abbreviations:* BMI: body mass index; PAI-1: plasminogen activator inhibitor-1; ICAM: intracellular adhesion molecule; IL-6: interleukin-6

**Table S3.** Results from latent class analysis for clinical and biomarker data

|  |  |  | **Number of Individuals per class** | | | | | |  |
| --- | --- | --- | --- | --- | --- | --- | --- | --- | --- |
|  | **BIC** | **Entropy** | **1** | **2** | **3** | **4** | **5** | **6** | **p-value*** |
| 1 Class | 24,341 |  | 543 (100%) |  |  |  |  |  |  |
| 2 Classes | 24,098 | 0.908 | 66 (12%) | 477 (88%) |  |  |  |  | 0.01 |
| 3 Classes | 24,022 | 0.732 | 65 (12%) | 166 (31%) | 312 (57%) |  |  |  | 0.18 |
| 4 Classes | 24,018 | 0.760 | 32 (6%) | 71 (13%) | 152 (28%) | 288 (53%) |  |  | 0.63 |
| 5 Classes | 24,031 | 0.765 | 32 (6%) | 52 (10%) | 55 (10%) | 161 (30%) | 243 (45%) |  | 0.34 |
| 6 Classes | 24,054 | 0.796 | 17 (3%) | 33 (6%) | 40 (7%) | 99 (18%) | 140 (26%) | 214 (39%) | 0.67 |

*P-value from VLMR test (adjusted for class number) and compares fit to model with 1 fewer class

*Abbreviations:* BIC: Bayesian information criterion

**Table S4.** Results from latent class analysis sensitivity analysis removing the highly correlated variables albumin, heart rate and urine output

|  |  |  | **Number of Individuals Per Class** | | | |  |
| --- | --- | --- | --- | --- | --- | --- | --- |
|  | **BIC** | **Entropy** | **1** | **2** | **3** | **4** | **p-value*** |
| 1 Class | 20,819 |  | 543 (100%) |  |  |  |  |
| 2 Classes | 20,573 | 0.912 | 63 (12%) | 480 (88%) |  |  | <0.01 |
| 3 Classes | 20,561 | 0.782 | 63 (12%) | 121 (22%) | 359 (66%) |  | 0.06 |
| 4 Classes | 20,556 | 0.811 | 23 (4%) | 58 (11%) | 108 (20%) | 354 (65%) | 0.59 |

*P-value from VLMR test (adjusted for class number) and compares fit to model with 1 fewer class

*Abbreviations:* BIC: Bayesian information criterion

**Table S5.** Biomarkers variables not included in latent class analysis (N=543)

| **Biomarker** | **Overall (n=543)** | **Phenotype 1**  **(n=66, 12%)** | **Phenotype 2**  **(n=477, 88%)** | **p-value** |
| --- | --- | --- | --- | --- |
| Ang2, pg/mL, median [IQR] | 8,389  [3,911 – 18,517] | 14,924  [6,368 – 34,681] | 7,982 [  3,660 – 16,716] | <0.01 |
| VCAM, ng/mL, median [IQR] | 1,942  [1,089 – 3,977] | 5,716  [3,969 - 7272] | 1,774  [1,045 – 3,215] | <0.01 |
| sFlt-1, pg/mL, median [IQR] | 269 [154 - 508] | 422 [232 - 892] | 253 [152 - 466] | <0.01 |
| E-Selectin, ng/mL, median [IQR] | 91 [56 - 200] | 134 [75 - 215] | 91 [55 - 194] | 0.24 |
| TNF, pg/mL, median [IQR] | 38 [18 - 120] | 124 [39 - 287] | 35 [17 - 91] | <0.01 |
| IL-10, pg/mL, median [IQR] | 25 [13 - 91] | 101 [22 - 966] | 20 [13 - 69] | <0.01 |
| Lactate, mmol/L, median [IQR]) | 2.5 [1.4 - 4.3] | 3.6 [1.7 - 7.1] | 2.4 [1.4 - 3.8] | <0.01 |
| D-Dimer, ng/mL, median [IQR] | 0.5 [0.5 - 1] | 0.5 [0.5 - 1] | 0.5 [0.5 - 1] | 0.17 |
| Urine Cr, mg/dL median [IQR] | 89 [50 - 150] | 90 [41 - 191] | 89 [51 - 148] | 0.65 |
| Urine Na, mEq/L, median [IQR] | 2.1 [1.5 - 3.1] | 3.2 [1.6 - 3.9] | 2.1 [1.4 - 2.9] | 0.06 |

*Abbreviations:* Ang2: angiopoietin-2; IQR: interquartile range; VCAM: vascular cell adhesion molecule; sFLT-1: soluble fms-like tyrosine kinase-1; TNF: tumor necrosis factor; IL-10: interleukin-10; Cr: creatinine; Na: sodium.

**Table S6.** Post-randomization processes of care by phenotype and treatment arm (N=364)

| **Treatment Arm** | | **Phenotype 1 (n=39)** | **Phenotype 2 (n=325)** | **p-value^b^** |
| --- | --- | --- | --- | --- |
| **Intravenous fluid volume^a^** | |  |  |  |
|  | EGDT, mL mean (SD) | 3,407 (1,668) | 2,578 (2,209) | 0.07 |
|  | Usual Care, mL mean (SD) | 3,215 (3,066) | 2,234 (1,956) | 0.28 |
| **Antibiotics^a^** | |  |  |  |
|  | EGDT, no. (%) | 26/26 (100%) | 152/159 (96%) | 0.28 |
|  | Usual Care, no. (%) | 13/13 (100%) | 162/166 (98%) | 0.57 |
| **Vasopressors^a^** | |  |  |  |
|  | EGDT, no. (%) | 22/26 (85%) | 86/159 (54%) | <0.01 |
|  | Usual Care, no. (%) | 6/13 (46%) | 79/166 (48%) | 0.92 |
| **Steroids^a^** | |  |  |  |
|  | EGDT, no. (%) | 9/26 (35%) | 36/159 (23%) | 0.19 |
|  | Usual Care, no. (%) | 3/13 (23%) | 29/166 (18%) | 0.61 |

**^a^**Intervention from time of randomization to 6 hours

**^b^**P-value for phenotype by treatment interaction derived from logistic regression models

*Abbreviations:* EGDT: early, goal-directed therapy

**Table S7.** Patient characteristics by treatment arm and phenotype (N=364)

|  | | **Phenotype 1 (n=39)** | | **Phenotype 2 (n=325)** | |
| --- | --- | --- | --- | --- | --- |
|  | | **EGDT** | **Usual Care** | **EGDT** | **Usual Care** |
| **N (%)** | | 26 (67%) | 13 (33%) | 159 (49%) | 166 (51%) |
| **Demographics** | |  |  |  |  |
|  | Age, years, mean (SD) | 64.0 (11.1) | 59.8 (15.1) | 58.8 (16.5) | 60.3 (15.8) |
|  | Gender, no. (%) |  |  |  |  |
|  | Male | 18 (69.2%) | 11 (84.6%) | 83 (52.2) | 97 (58.4%) |
|  | Female | 8 (30.8%) | 2 (15.4%) | 76 (47.8) | 69 (41.6%) |
|  | Race, no. (%) |  |  |  |  |
|  | White | 20 (76.9%) | 7 (53.8%) | 109 (68.6%) | 121 (72.9%) |
|  | Black | 4 (15.4%) | 4 (30.8%) | 34 (21.4%) | 35 (21.1%) |
|  | Other | 2 (7.7%) | 7 (15.4%) | 16 (10.0%) | 10 (6.0%) |
|  | Apache III score, mean (SD) | 66.8 (26.1) | 68.5 (23.2) | 59.9 (21.9) | 61.5 (22.8) |
| **Clinical Characteristics** | |  |  |  |  |
|  | Albumin, g/dL, mean (SD) | 2.8 (0.8) | 2.7 (0.7) | 3.2 (0.8) | 3.1 (0.9) |
|  | BMI, kg/m^2^, mean (SD) | 27.7 (8.7) | 28.5 (8.0) | 28.2 (7.4) | 28.2 (8.5) |
|  | Serum Creatinine, mg/dL, mean (SD) | 2.0 (1.1) | 2.1 (1.9) | 2.3 (2.0) | 2.4 (2.1) |
|  | Glucose, mg/dL, mean (SD) | 110.5 (51.5) | 140.8 (71.5) | 168.4 (129.5) | 172.0 (135.6) |
|  | Heart Rate, beats/min, mean (SD) | 113.3 (24.9) | 113.8 (19.9) | 110.9 (23.8) | 112.2 (23.9) |
|  | Hematocrit, %, mean (SD) | 30.7 (8.0) | 33.0 (5.8) | 36.5 (7.3) | 35.2 (6.7) |
|  | Platelet Count, in thousands, mean (SD) | 67.8 (56.5) | 59.4 (49.2) | 228.7 (118.4) | 252.7 (144.5) |
|  | Respiratory Rate, resps/min, mean (SD) | 23.7 (7.2) | 20.2 (7.1) | 22.4 (6.5) | 22.9 (7.0) |
|  | Systolic Blood Pressure, mmHg, mean (SD) | 97.9 (29.0) | 105.8 (29.8) | 100.4 (29.2) | 98.3 (28.7) |
|  | Sodium, mEq/L, mean (SD) | 135.2 (4.6) | 135.9 (7.1) | 135.6 (5.9) | 135.2 (6.9) |
|  | Temperature, °C, mean (SD) | 37.3 (1.7) | 37.2 (1.8) | 37.3 (1.5) | 37.5 (1.6) |
|  | Total Bilirubin, mg/dL, mean (SD) | 5.1 (3.2) | 4.2 (3.6) | 1.0 (0.5) | 1.2 (1.4) |
|  | Vasopressors, no. (%) | 3 (11.5%) | 3 (23.1%) | 37 (23.2%) | 25 (15.1%) |
|  | Mechanical Ventilation, no. (%) | 5 (19.2%) | 3 (23.1%) | 31 (19.5%) | 19 (11.5%) |

*Abbreviations:* EGDT: early, goal-directed therapy; SD: standard deviation

**Table S8.** Treatment effect on clinical outcomes by phenotype (N=364)

| **Treatment Arm** | | **Phenotype 1 (n=39)** | **Phenotype 2 (n=325)** | **p-value*** |
| --- | --- | --- | --- | --- |
| **60-day inpatient mortality** | |  |  |  |
|  | EGDT, no. (%) | 15/26 (58%) | 25/159 (16%) | 0.05 |
|  | Usual Care, no. (%) | 3/13 (23%) | 28/166 (17%) |  |
| **365-day mortality** | |  |  |  |
|  | EGDT, no. (%) | 20/26 (77%) | 56/159 (35%) | 0.13 |
|  | Usual Care, no. (%) | 7/13 (54%) | 62/166 (37%) |  |

*P-value for phenotype by treatment interaction derived from logistic regression models

*Abbreviations:* EGDT: early, goal-directed therapy

**Table S9.** Short- and long-term outcomes for treatment by APACHE III score (N=364)

| **Treatment Arm** | | **EGDT (n=185)** | **Usual Care (n=179)** | **p-value*** |
| --- | --- | --- | --- | --- |
| **60-day inpatient mortality** | |  |  |  |
|  | Mortality, no. (%) | 40/185 (22%) | 31/179 (17%) | 0.42 |
|  | Apache III, mean (SD) | 61 (23) | 62 (23) |  |
| **365-day mortality** | |  |  |  |
|  | Mortality, no. (%) | 76/185 (41%) | 69/179 (39%) | 0.34 |
|  | Apache III, mean (SD) | 61 (23) | 62 (23) |  |

*P value for phenotype by treatment interaction derived from logistic regression models

*Abbreviations:* EGDT: early, goal-directed therapy

**Table S10.** Clinical characteristics and outcomes of phenotype 1 and SENECA delta phenotype

| **Variable** | | **ProCESS**  **phenotype 1**  **(n=66, 12%)** | **SENECA 8**  **phenotype (n=89, 16%)** |
| --- | --- | --- | --- |
| **Demographics** | |  |  |
|  | Age, years, mean (SD) | 61 (13) | 64.3 (14.0) |
|  | Gender, no. (%) |  |  |
|  | Male | 47 (71.2%) | 58 (65%) |
|  | Female | 19 (28.8%) | 31 (35%) |
|  | Race, no. (%) |  |  |
|  | White | 44 (66.7%) | 57 (64%) |
|  | Black | 15 (22.7%) | 24 (27%) |
|  | Other | 7 (10.6%) | 8 (9%) |
|  | Charlson Comorbidity Index, mean (SD) | 3.2 (2.7) | 2.9 (2.8) |
|  | Apache III score, mean (SD) | 69.6 (22.2) | 80.7 (28.8) |
| **Clinical Variables** | |  |  |
|  | Albumin, g/dL, mean (SD) | 2.7 (0.8) | 2.6 (0.9) |
|  | Serum Creatinine, mg/dL, mean (SD) | 2.1 (1.4) | 3.0 (1.9) |
|  | C-reactive protein, mg/L, mean (SD) | 132.7 (139.8) | 147.9 (133.4) |
|  | Heart Rate, beats/min, mean (SD) | 116 (23) | 115.6 (23.9) |
|  | Hematocrit, %, mean (SD) | 31 (7) | 36.3 (7.9) |
|  | Lactate, mmol/L, mean (SD) | 4.7 (3.7) | 5.9 (4.3) |
|  | Platelet Count, in thousands, mean (SD) | 73 (55) | 187.9 (127.1) |
|  | Respiratory Rate, resps/min, mean (SD) | 22 (7) | 24.7 (9.7) |
|  | Systolic Blood Pressure, mmHg, mean (SD) | 102 (30) | 94.5 (28.3) |
|  | Total Bilirubin, mg/dL, mean (SD) | 4.8 (3.5) | 2.6 (3.1) |
|  | WBC Count, in thousands, mean (SD) | 5 (6) | 17.0 (11.5) |
|  | Vasopressors, no. (%) | 15 (23%) | 25 (28%) |
| **Outcomes** | |  |  |
|  | Admission to Intensive Care Unit, no. (%) | 64 (97%) | 87 (98%) |
|  | 60-day Inpatient Mortality, no. (%) | 27 (41%) | 36 (40%) |
|  | 365-day Mortality, no. (%) | 44 (67%) | 53 (60%) |

**Table S11.** Biomarker comparison in clinical + biomarker phenotypes to clinical only phenotypes

| **Biomarker** | **Phenotype 1** | **Phenotype 2** | **SENECA 8**  **Phenotype** | **SENECA Non-**  **8 Phenotype** | **p-value** |
| --- | --- | --- | --- | --- | --- |
| N (%) | 66 (12%) | 477 (88%) | 89 (16%) | 454 (84%) |  |
| ICAM, ng/mL,  median [IQR] | 1320  [607 - 2425] | 466  [310 - 683] | 582  [385 - 1051] | 480  [315 - 752] | <0.01 |
| IL-6, pg/mL, median [IQR] | 4841  [419 - 57,464] | 281  [77 - 1774] | 1407  [223 - 9043] | 291  [72 - 2307] | <0.01 |
| PAI-1, ng/mL,  median [IQR] | 14  [8 - 37] | 15  [8 - 27] | 20  [10 - 40] | 14  [7 - 27] | 0.49 |

*Abbreviations:* ICAM: intracellular adhesion molecule; IQR: interquartile range; IL-6: interleukin- 6; PAI-1: plasminogen activator inhibitor-1
